# Damage Evolution in Complex-Phase and Dual-Phase Steels during Edge Stretching

**DOI:** 10.3390/ma10040346

**Published:** 2017-03-27

**Authors:** Nikky Pathak, Cliff Butcher, Michael James Worswick, Erika Bellhouse, Jeff Gao

**Affiliations:** 1 Department of Mechanical Engineering, University of Waterloo, Waterloo, ON N2L 3G1, Canada; cbutcher@uwaterloo.ca (C.B.); worswick@lagavulin.uwaterloo.ca (M.J.W.); 2 Research and Development, ArcelorMittal Dofasco, Hamilton, ON L8H 3N8, Canada; erika.bellhouse@arcelormittal.com (E.B.); jeff.gao@arcelormittal.com (J.G.)

**Keywords:** ductile failure, damage, voids, dual-phase steel, ferritic-bainitic steel

## Abstract

The role of microstructural damage in controlling the edge stretchability of Complex-Phase (CP) and Dual-Phase (DP) steels was evaluated using hole tension experiments. The experiments considered a tensile specimen with a hole at the center of specimen that is either sheared (sheared edge condition) or drilled and then reamed (reamed edge condition). The damage mechanism and accumulation in the CP and DP steels were systematically characterized by interrupting the hole tension tests at different strain levels using scanning electron microscope (SEM) analysis and optical microscopy. Martensite cracking and decohesion of ferrite-martensite interfaces are the dominant nucleation mechanisms in the DP780. The primary source of void nucleation in the CP800 is nucleation at TiN particles, with secondary void formation at martensite/bainite interfaces near the failure strain. The rate of damage evolution is considerably higher for the sheared edge in contrast with the reamed edge since the shearing process alters the microstructure in the shear affected zone (SAZ) by introducing work-hardening and initial damage behind the sheared edge. The CP microstructures were shown to be less prone to shear-induced damage than the DP materials resulting in much higher sheared edge formability. Microstructural damage in the CP and DP steels was characterized to understand the interaction between microstructure, damage evolution and edge formability during edge stretching. An analytical model for void evolution and coalescence was developed and applied to predict the damage rate in these rather diverse microstructures.

## 1. Introduction

Advanced high strength steels (AHSS), in particular dual-phase (DP) steels, have seen widespread adoption for automotive body-in-white and chassis applications due to their high strength and good formability. DP steels derive their strength and ductility from a multiphase microstructure which comprises a high-strength martensitic phase within a soft ferritic matrix [1]. These steels exhibit good in-plane formability, as characterized by a forming limit diagram approach, for example. However, they are susceptible to premature failure during industrial stretch-flanging operations and often exhibit failure strains at the sheared edge that are below the forming limit strain [2,3]. Good stretch-flangeability is an essential property for forming complex press-formed parts such as chassis, suspension arm and control arm components, making DP steels less suitable for edge stretching operations. Consequently, there is a great interest in developing materials that could improve the performance of AHSS in industrial stretch-flangeable operations.

As a potential alternative to dual-phase steels, previous researchers [4,5,6,7], reported that bainitic microstructures can offer improved sheared edge stretchability. The performance of ferritic-bainitic steels has been further enhanced by the development of multi-phase hot-rolled stretch-flangeable complex phase (CP) steels that consist of mainly ferrite and bainite with a minor fraction of martensite [6]. Recently, Pathak et al. [7] investigated CP800 steels with strength of 800 MPa and reported edge stretchability to be three times larger than that of DP780 of similar strength. These investigations suggest that ferritic-bainitic steels, especially CP steels, could be promising for automotive components that require high sheared edge stretch-flangeability.

The shearing process introduces two zones; a shear face (sheared edge) and a zone of deformation behind the shear face known as the shear-affected zone (SAZ) [8]. The shear face is comprised of four zones: roll-over, burnish, fracture, and shear-burr [8]. The SAZ is characterized by severe work-hardening and damage in the form of pre-nucleated voids formed during the shearing process. In the automotive industry, holes are generally sheared because it is more economical and faster than machining operations. However, the presence of the SAZ reduces the formability of the sheared edge [8,9,10,11]. Since the features of the sheared edge are formed as a result of deformation and fracture within the material, the sensitivity to the shearing process during subsequent edge stretching is strongly dependent upon the microstructure.

For high strength-low alloy (HSLA) and mild steels, the sheared edge stretchability is affected by the presence of inclusions, with lower sulfide content and low inclusion aspect ratios leading to better edge formability [12]. Adamczyk and Michal [13] found that steel alloys with rare earth elements such as lanthanum showed better performance in sheared edge stretching compared to alloys that did not contain rare earth elements. Microstructural compositions that contribute to better sheared edge quality translate into improved edge stretchability. The sheared edge stretchability of AHSS is even more strongly dependent on the microstructure due to the presence of multiple phases and high strength differential between phases [14,15,16]. Hasagwa et al. [14] suggested that the relative strength differential between ferrite and martensite was the driving mechanism for reduced edge stretchability in DP steels. Kumar et al. [15] and Sudo et al. [16] reported higher stretch-flangeability for ferritic-bainitic DP steels due to the lower relative hardness between the ferrite and bainite phases. This difference in microstructural properties leads to variation in failure mechanism and consequently alters the edge stretchability of a material. The fracture mechanism during sheared edge stretching has been rarely investigated. Teng and Chen [17] examined the fracture mechanism behind reamed and sheared edges qualitatively, however the underlying damage parameter influencing formability of the sheared edge has remained unknown.

A typical ductile failure is divided into three stages comprising void nucleation, growth, and coalescence. In a homogeneous material such as ferritic steels, void nucleation is associated with either non-metallic inclusions (such as manganese sulfide and calcium containing inclusions) [18,19] or carbide particles (such as titanium carbides, iron carbides, chromium carbides) [20,21]. Wallin et al. [22] reported carbide diameter to be a major factor controlling fracture in bainitic steels. A lower carbon content promotes ductile failure in martensitic steels while intergranular or transgranular fracture occurs at higher carbon content [23]. The failure mechanism in AHSS steels was found to be different than that in conventional and mild steels due to their complex multiphase microstructure [1]. Numerous work has been done to characterize damage mechanisms in ferritic steels [18,19,20,21], martensitic steel [23,24,25], bainitic steel [22], and ferritic-martensitic DP steel. Different void nucleation modes reported for DP steels are (a) decohesion of ferrite-martensite interface [26]; (b) martensite cracking [27]; (c) debonding of ferrite grain boundaries [28]; and (d) debonding or fracture of inclusions [29]. The former two are the most prominent modes of nucleation modes observed in DP steels. It was reported that the morphology of martensite plays a prominent role in influencing the strength and formability in DP steels [30]. Recently, Kahziz et al. [31] assessed damage evolution during simultaneous loading and bending of a DP600 sheared edge using laminography. However, the available studies on AHSS grades have mostly focused on the failure mechanism under tensile or proportional loading. The work done to characterize the failure mechanism in ferritic-bainitic steels is limited to examination of the fracture surface. Metallographic studies on initiation and quantification of damage processes within ferritic-bainitic steels have not been reported to-date. The investigation of damage mechanisms under non-proportional loading such as sheared edge stretching has not received much attention to-date, but is necessary for the identification of microstructural parameters required to simulate this process.

The current work addresses this need to investigate the role of the microstructure and damage mechanism in two AHSS of the same strength level and to quantify the relationship between damage process and edge condition. Traditionally, a hole expansion test is used to determine edge failure which consists of expanding a hole with a conical punch until failure. The edge stretchability is dependent on the punch geometry and orientation of burr during the hole expansion test [7]. In an alternate test to observe failure during edge stretching, a hole was fabricated at the center of tensile specimens that were interrupted at different strain levels. Unlike the hole expansion test, there is no influence of burr-orientation or punch geometry on the edge stretching response during the hole tension test and therefore this test was selected for the present study. Two different edge conditions, corresponding to drilled and reamed versus sheared holes, were considered for this study to isolate the effect of pre-straining and damage nucleation during the shearing process on damage evolution during edge stretching. A series of interrupted tests using stereo digital image correlation was used to obtain the local strain at the edge and then used to directly correlate the edge strain with void measurements and source of damage obtained at the same location using optical microscopy and scanning electron microscope (SEM) respectively. In this novel approach, void nucleation, evolution, and coalescence can be directly correlated with the local strain obtained for sheared and reamed edges as well as the microstructure. The quantitative data obtained in this work was used to quantify the effect of the large shear strains and damage nucleation occurring during edge shearing on subsequent damage evolution during hole expansion. Measurements of void nucleation, growth, and coalescence were used to implement the Chu and Needleman [32] criterion for nucleation, the Rice and Tracey [33] model for void growth, and the Benzerga and Leblond [34] model for void coalescence to describe the inter-relationship between damage mechanisms and edge stretching limits. This correlation between damage parameters and sheared edge formability is important in order to quantify the relationship between microstructure and sheared edge stretching performance as well as to understand the interaction between residual shear strain field and damage evolution. An investigation into these interrelationships is needed to contribute to future work to develop a suitable fracture model for finite element simulations of edge stretching.

## 2. Experimental Procedure

### 2.1. Material Characterization

**Figure 1:**
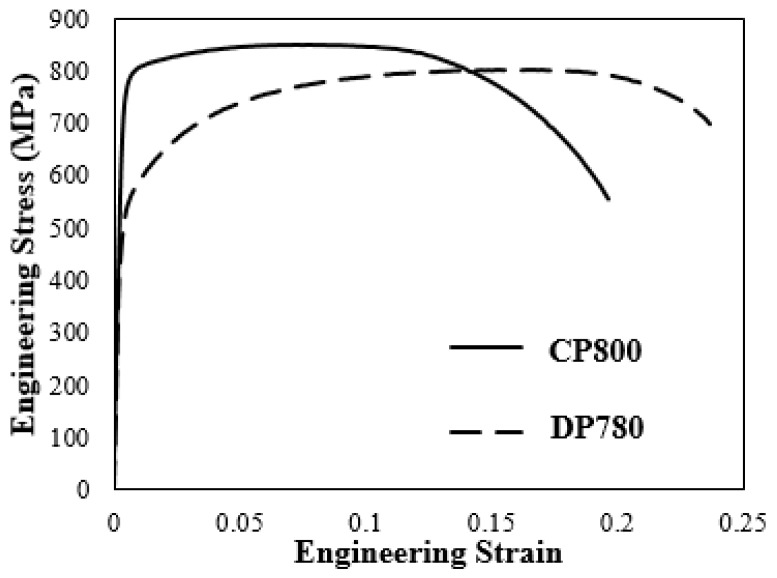
Engineering stress-strain curves (TD) for the CP and DP steels at 0.003 s^−1^ strain rate [7].

The present work focuses on the CP800 and DP780 steels, the chemical compositions of which are shown in Table 1. The materials investigated herein have been characterized by the present authors using sub-size ASTM (ASTM E8M-11) tensile specimens in [7]. In that work, experiments were performed along the rolling (RD), diagonal (DD), and transverse directions (TD) of the sheet at a strain rate of 0.003 s^−1^ using a gauge length of 25 mm. Subsize ASTM (ASTM E8M-11) tensile specimens for the CP800 and DP780 steels were tested along the rolling (RD), diagonal (DD), and transverse directions (TD) of the sheet at a strain rate of 0.003 s^−1^ using a gauge length of 25 mm [7]. The relevant mechanical properties of the CP and DP steels such as yield strength (YS), ultimate tensile strength (UTS), percentage total elongation (% TE), strain hardening exponent (*n*), Lankford coefficients (*r*), normal anisotropy coefficient (*r_n_*), planar anisotropy coefficient (*r_a_*), and the percentage reduction of area (% RA) are listed in Table 2. The engineering stress-strain curves for each material in the transverse direction are shown in Figure 1. It is worth noting that while the UTS of DP780-designated materials is fixed at 780 MPa (or better), there can be significant variation in yield strength. For example, the DP780 sheet studied by Kadkhodapour et al. [28] has a UTS of 795 MPa and YS of 480 MPa, whereas the DP780 investigated in the current work has a UTS and YS of 800 MPa and 509 MPa, respectively.

**Figure 2:**
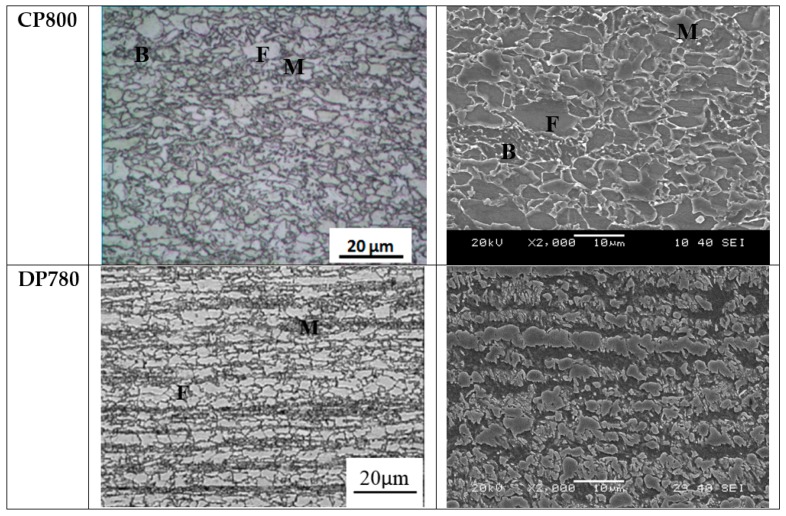
Optical and scanning electron microscopy (SEM) micrographs of the CP800 and DP780 with martensite (**M**), bainite (**B**), and ferrite (**F**) microstructures indicated.

Undeformed samples of the as-received steel sheet were mounted in epoxy and polished to a mirror finish. The samples were etched with Nital (5% nitric acid by volume) to reveal the grain boundaries and analyzed using an optical microscope and a SEM. Figure 2 shows the micrographs of the CP800 and DP780 steels. The CP800 alloy is mainly composed of ferrite and bainite with a minor fraction of martensite. Bell [35] performed quantitative metallographic examination of the same lot of CP800 sheet and reported the presence of 1%–2% martensite. Winkler et al. [36] previously characterized the same DP780 material used in this study and reported that DP780 contains approximately 63% ferrite with the balance composed of martensite and bainite. In the optical micrograph, the lighter grey regions are ferrite and the darker grey regions are bainite and martensite. Ferrite reacts after application of a Nital etch and martensite remains unaffected, such that ferrite appears as the darker phase and martensite appears as a brighter phase, as indicated in Figure 2 [37]. The bainite manifests as a ferrite matrix with martensite islands. A darker ferrite matrix with islands of brighter martensite is bainite as shown in Figure 2 [37]. The DP780 consists of a ferrite-martensite microstructure with a small fraction of bainite and bands of martensite in the rolling direction.

### 2.2. Hole Processing Techniques

The different hole processing techniques used in the present study: (i) drilled and reamed; and, (ii) sheared, are discussed in the following sections.

#### 2.2.1. Reamed Hole

The reamed holes were processed by first drilling a pilot hole of 9.5 mm diameter, followed by reaming to a diameter of 10 mm. The surface was lightly polished with 300 grit SiC paper to remove any surface roughness or drill-burr at the edge. This reamed and polished edge is considered to have minimal work-hardening relative to the sheared holes and therefore can be represented as an “ideal hole” that can directly relate formability to the microstructure of the material.

#### 2.2.2. Sheared Hole

**Figure 3:**
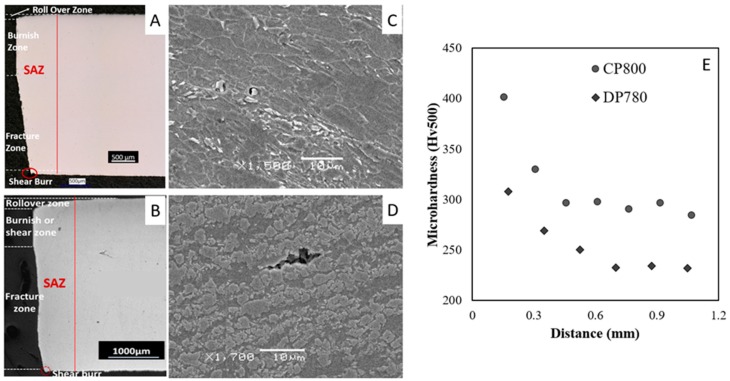
Sheared edge of CP800 (**A**); and DP780 (**B**); consisting four zones: rollover, burnish, fracture, and burr. Pre-nucleated voids are found in the shear-affected-zone away from the CP800 (**C**); and DP780 (**D**) sheared edge; (**E**) the hardness profile for the two sheared edges in the rolling direction.

The hole punching process followed in the authors previous work [7] was used in the current study to maintain the same hole quality i.e., punched holes were sheared with a 12% die clearance using an 85 ton Die Master punch press. Figure 3A shows a micrograph of the sheared edge, with the roll-over, burnish, fracture zones, and shear-burr indicated. A new punch was used to ensure consistent hole quality and mitigate burr formation. Void nucleation resulting from the shearing process is evident in Figure 3B. The hardness profile of the sheared edge (Figure 3C) indicates the presence of work-hardening that reaches a maximum at the edge and then decreases to the hardness of the undeformed material. Based upon these hardness measurements, the size or depth of the SAZ of the DP780 and CP800 samples is about 41% and 20% of the sheet thickness, respectively.

### 2.3. Hole Tension Test

**Figure 4:**
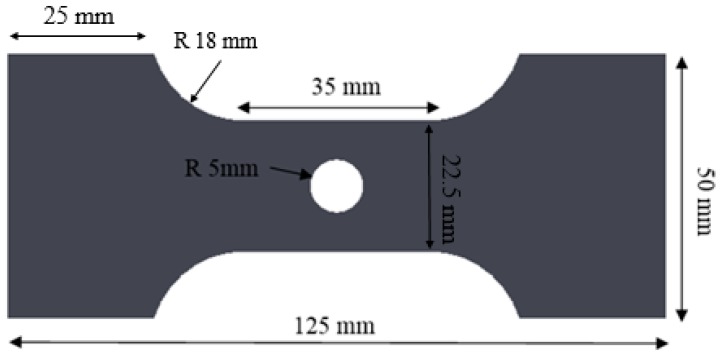
A specimen geometry of DP780 hole tension test.

A tensile specimen with a gauge length of 35 mm was fabricated with a central hole of diameter 10 mm, as shown in Figure 4. The ratio of the width of the ligament on either side of the hole to the sheet thickness is 4.2 for the two materials investigated. The specimen was subjected to tension in a servo hydraulic Instron testing apparatus at a cross-head displacement of 0.075 mm/s. In earlier hole expansion experiments using a conical punch by Pathak et al. [7], fracture occurred along the rolling direction under tensile stresses acting along the transverse direction. Thus, the transverse loading direction was selected in the present study to replicate failure conditions during the hole expansion test. A stereo digital image correlation (DIC) system was used to record the full-field strain during the experiment using an acquisition rate of 4 images per second and image size of 2448 × 2048 pixels. The DIC algorithm [38] utilized a step size, and filter size of 4 and 13 pixels, respectively were used at a resolution of 0.02 mm/pixel. A vertical virtual extensometer of length 18 mm was used to measure the displacement to failure. A circle of diameter 0.25 mm was used to extract DIC strain measurements and an equivalent strain was calculated based on Von Mises theory and volume conservation as shown in Equation (1). The hole tension tests were interrupted at different strain levels to systematically characterize the damage mechanism and the accumulation in the CP and DP steels.

(1) $$\varepsilon_{eq}=\frac{2}{\sqrt{3}}\sqrt{\left(\varepsilon_{1}^{2}+\varepsilon_{2}^{2}+\varepsilon_{1}^{2}\varepsilon_{2}^{2}\right)}$$

### 2.4. Metallographic Specimen Preparation

**Figure 5:**
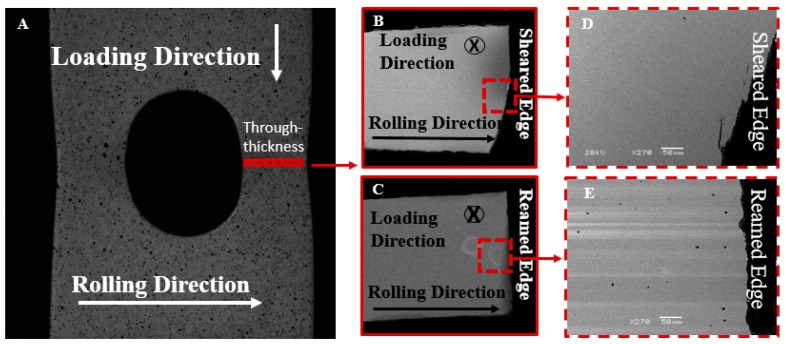
(**A**) A hole tension specimen indicating the area observed under SEM behind the CP800 sheared; and reamed edges indicated in (**B**,**C**) respectively; The magnified views behind the two edges are shown in (**D**,**E**).

The characterization of damage accumulation was performed on interrupted hole tension specimens using optical and scanning microscopy. One half of the specimen was sectioned along the specimen mid-plane (orthogonal to the loading direction (indicated in Figure 5A), after which it was mounted and polished to a mirror surface. The surface was etched with Nital (5% volume) to reveal grain boundaries and to determine phases under SEM, as shown in Figure 5B,C for the sheared and reamed edges respectively. The measured strain field from the in situ DIC analysis was overlaid on the optical micrographs in order to relate observed microstructural damage to applied strain.

**Figure 6:**
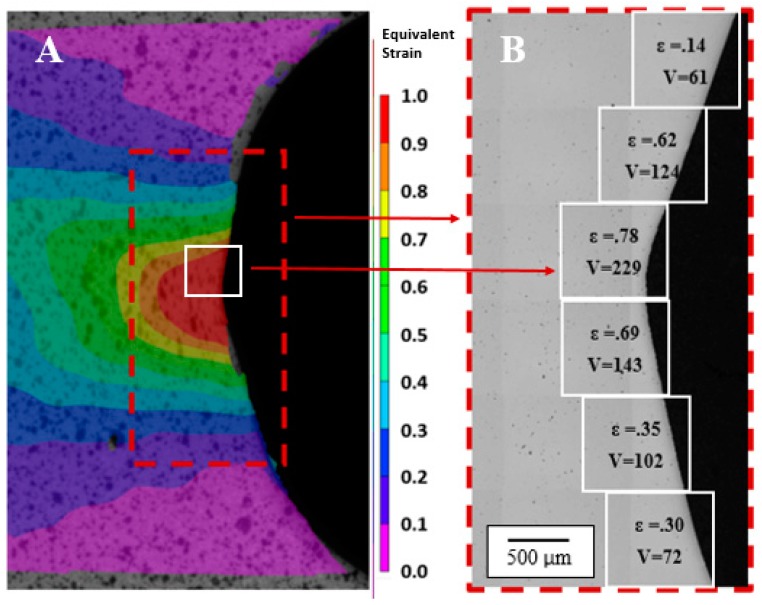
Contour plot of the equivalent strain and in-plane optical image of the CP800 reamed edge divided into six different strain levels along the corresponding optical image (**A**); Equivalent strain and number of voids (*V*) within each AOI are indicated (**B**).

The other half of the interrupted specimens was oriented in-plane and the same polishing procedure was applied as for the through-thickness specimen, followed by final polishing with colloidal silica. The DIC strain contour plot from the final image recorded during the hole tension test is shown in Figure 6A. The corresponding in-plane micrographs of the as-polished surface were captured using optical microscopy to reveal the void distribution. For each specimen, the optical image was acquired at 50× magnification and assembled as a mosaic image, as shown in Figure 6B for a CP800 reamed edge. The strain plot and optical image were divided into six square areas of interest (AOI) regions of approximately 1 mm along the edge, as indicated in Figure 6, from which the damage accumulation was measured. The strain for each AOI was determined from the DIC analysis using a corresponding gauge length of 1 mm. The equivalent strain, *ε* and number of voids, *V*, for each AOI are indicated in Figure 6B. In this manner, six data measurements (of damage versus strain level) were acquired from each specimen to quantify damage evolution in the DP780 and CP800 steels. The void data was quantified using the Image-Pro Version 5.0 software to provide measures of individual void characteristics within a square section. A set of pixels has to be statistically significant in two dimensions to be registered as a void; therefore, a void is included into analysis only when the diameter of the void exceeds twice the pixel size i.e., 2.0 µm.

## 3. Edge Stretchability of the CP and DP steels

**Figure 7:**
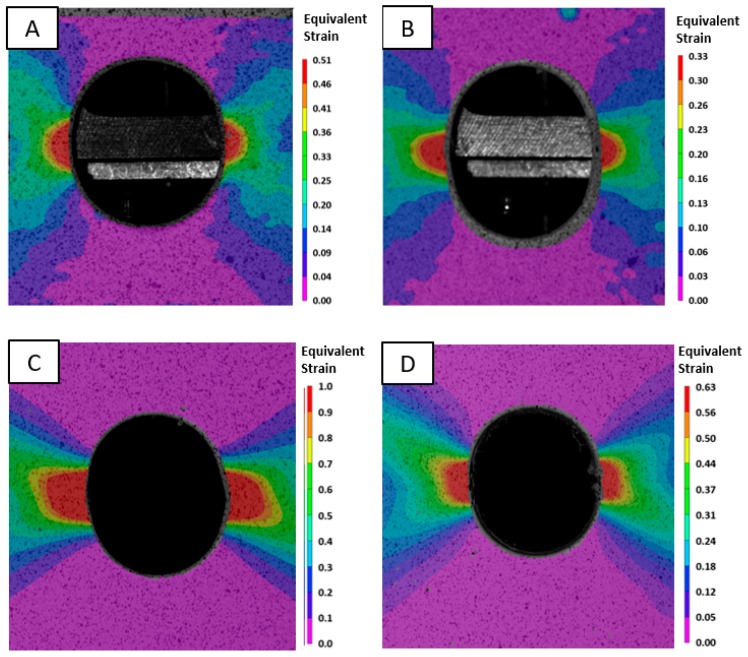
Contour plot of equivalent strain during the hole tension test of the (**A**) DP780 reamed edge; (**B**) DP780 sheared edge; (**C**) CP800 reamed edge; and (**D**) CP800 sheared edge.

**Figure 8:**
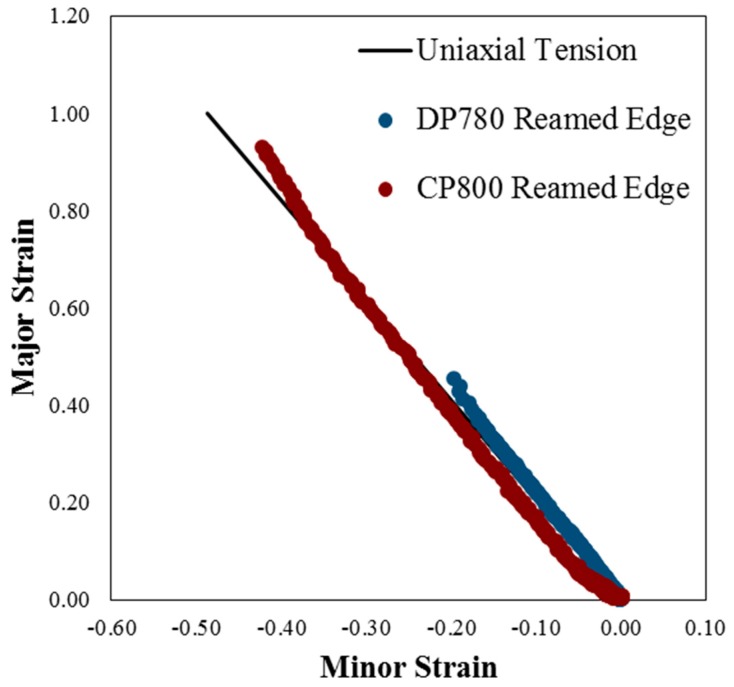
Strain-path for the deformation of CP800 and DP780 reamed edges during the hole tension test.

**Figure 9:**
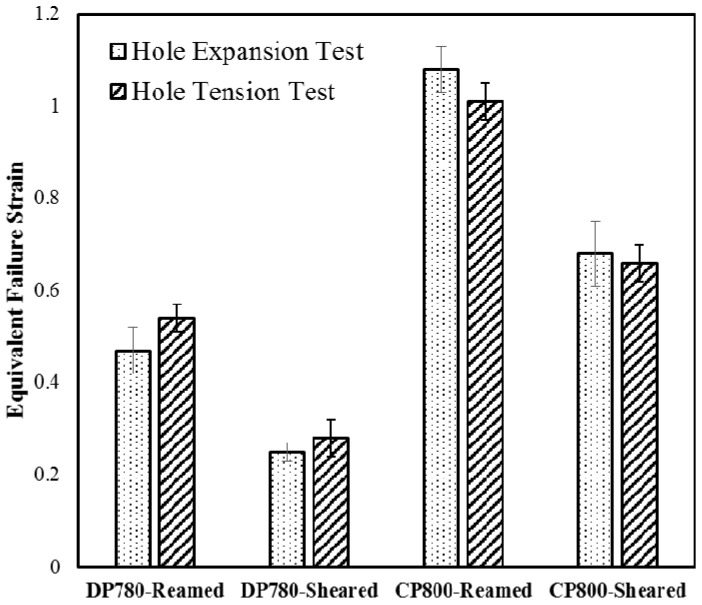
Equivalent failure strain for the DP780 and CP800 obtained during the current hole tension and hole expansion tests for the reamed versus sheared edges.

Table 3 indicates displacement to failure and fracture strain for the reamed and sheared edge conditions for the CP800 and DP780 steels. Figure 7 shows the contour plot of logarithmic equivalent strain calculated by 3D DIC analysis. The maximum deformation occurs along the specimen central plane at the hole edge and the crack is observed to be initiated from this region. Figure 8 shows the strain-path extracted near the hole edge at a location where a crack initiates during the hole tension test. The strain ratio for uniaxial tension corresponds to −0.5 for an isotropic material which changes based on the R-value of the material and is indicated in Figure 8, from which it is seen that the strain-path during the hole tension test follows a near uniaxial tensile loading condition until the onset of failure [39]. The local equivalent strain at failure in the hole tension experiments is plotted in Figure 9. It is evident that the CP sheet exhibits a much higher edge formability (equivalent strain to failure) than the DP alloy. The effect of shearing in reducing edge formability is also evident for both alloys. The results obtained for the DP780 reamed and sheared edges are consistent with the findings of the previous work [39] where it was found that the sheared edge has a much lower fracture limit than the reamed edge.

The measured strain-path during the hole tension test is close to uniaxial tension which is similar to the stress-state near the hole edge during a hole expansion test [39]. Hole expansion testing of the same lot of CP and DP steels was performed by Pathak et al. [7]. Approximately 40%–60% reduction in the hole expansion ratio was observed for the materials considered when a sheared hole was expanded, compared to a reamed hole. Butcher et al. [40] developed an analytical method to determine the equivalent failure strain in a hole expansion test, using the measured inner and outer hole diameters at fracture as well as the thickness around the circumference as:

(2) $$\varepsilon_{eq}^{macroscopic}=\frac{2}{3}\left(\varepsilon_{c}-\varepsilon_{t}\right)$$

(3) $$\varepsilon_{c}=\ln\left(\frac{d_{outer}-d_{inner}}{2\;d_{o}}\right)$$

(4) $$\varepsilon_{t}=\ln\left(\frac{t_{edge}}{t_{o}}\right)$$

When the shear burr is in the up position, it does not contact the punch and experiences pure uniaxial tension which is equivalent to the stress-state during hole tension testing. The equivalent strains obtained in the burr-up position during the hole expansion test and the failure strains for the reamed edge condition for each alloy are plotted in Figure 9 along with the current hole tensile results. The confidence intervals of failure strains for the different conditions obtained from the two tests overlap, suggesting there is no significant difference between the edge limit-strains determined from the two tests. This observation supports the use of the hole tension test as an alternative to the hole expansion test.

**Figure 10:**
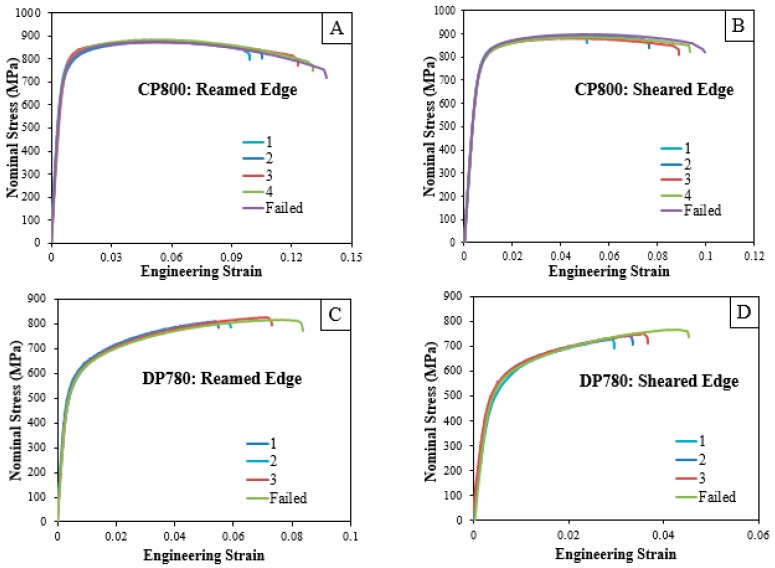
Histories of nominal stress versus equivalent strain for the (**A**) CP800 reamed; (**B**) CP800 sheared; (**C**) DP780 reamed and (**D**) DP780 sheared specimens interrupted at different strains.

To systematically characterize damage accumulation behavior in the DP780 and CP800 steels, hole tension tests were interrupted at three or four levels of displacement, respectively, for each edge condition as indicated in Table 3. Since the failure strain for the DP780 alloy was the lower of the two, the DP780 experiments were interrupted at three different strains, instead of four. The plots of nominal stress-engineering strain data for the sheared and reamed edges of the CP800 and DP780 specimens interrupted at different strain levels are shown in Figure 10. The nominal stress was calculated by dividing the load with the minimum cross-section area. The engineering strain was calculated using a virtual extensometer of length 18 mm.

## 4. Damage Development Resulting from Shearing and Hole Tensile Deformation

Successive micrographs of the hole edge deformed at various strain levels during the hole tension experiments were analyzed to examine the fracture mechanism in DP780 and CP800. The influence of shearing process on the damage mechanism was investigated by conducting SEM analysis on the through-thickness orientation for the reamed and sheared edges.

### 4.1. Fracture Mechanism in DP780

**Figure 11:**
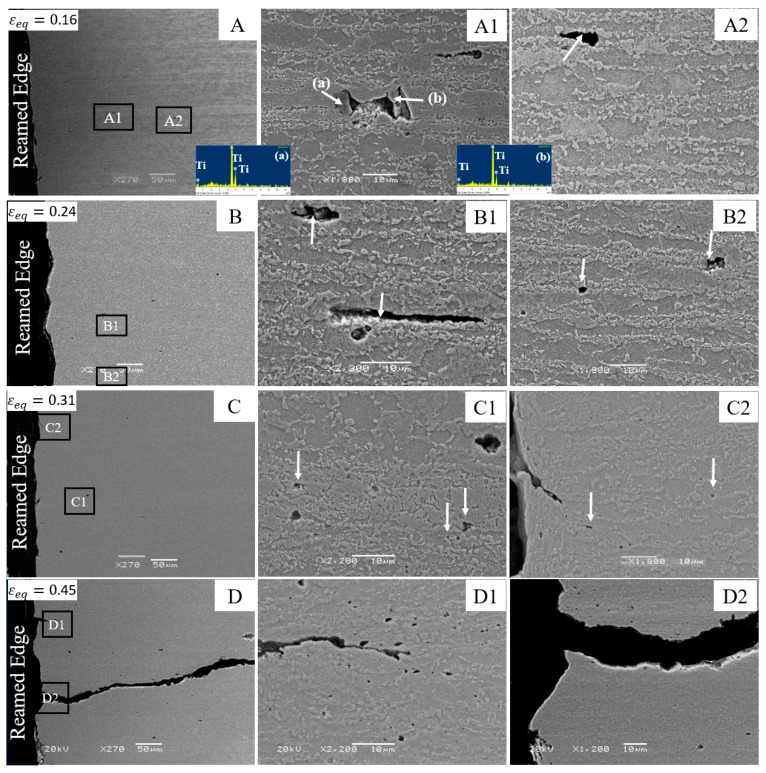
SEM micrographs of the interrupted DP780 reamed edge hole tension specimens at different strain levels: (**A**) 0.16; (**B**) 0.24; (**C**) 0.31; and (**D**) 0.45.

Figure 11 shows the progression of damage behind the DP780 reamed edge during hole tension deformation. The micrographs A, B, C, and D were acquired at the three different strain levels where deformation was interrupted. The local equivalent strains corresponding to each micrograph are 0.16, 0.24, 0.31, and 0.45, respectively (note that the last micrograph/strain level corresponds to the conditions at fracture). Micrograph A1 shows voids nucleated by de-bonding of the ferrite matrix from TiN particles and subsequent coalescence. The presence of two inclusions in close proximity leads to coalescence at a lower strain. Void nucleation due to the fracture of martensite is shown in micrograph A2. The DP780 SEM micrographs B1 and B2 reveal that voids mainly nucleate due to martensite cracking or debonding of the martensite-ferrite interface during deformation. At a higher strain of 0.31, void nucleation by de-cohesion of ferrite-martensite interfaces (C1) becomes the more dominant mechanism. A similar mechanism was suggested by Han and Margolin [41] who reported that void nucleation at martensite islands occurs at lower strain while the voids associated with ferrite-martensite interface decohesion nucleate closer to the fracture strain. Micrograph C2 indicates the fourth category of void nucleation by failure of ferrite grains along with the crack initiation at the edge. A typical fractured DP780 reamed edge is shown in micrograph D. The rate of void nucleation with strain is initially low and increases closer to final failure. More voids form in the vicinity of the crack, as shown in micrograph D1, presumably due to the enhanced stress triaxiality near the crack-tip. The propagation of the crack eventually occurs by linking of voids and results in the fracture of the DP780 reamed edge, as shown in micrograph D2. An irregular crack-path is associated with void nucleation, growth, and coalescence and indicates ductile failure in the DP780 reamed edge.

**Figure 12:**
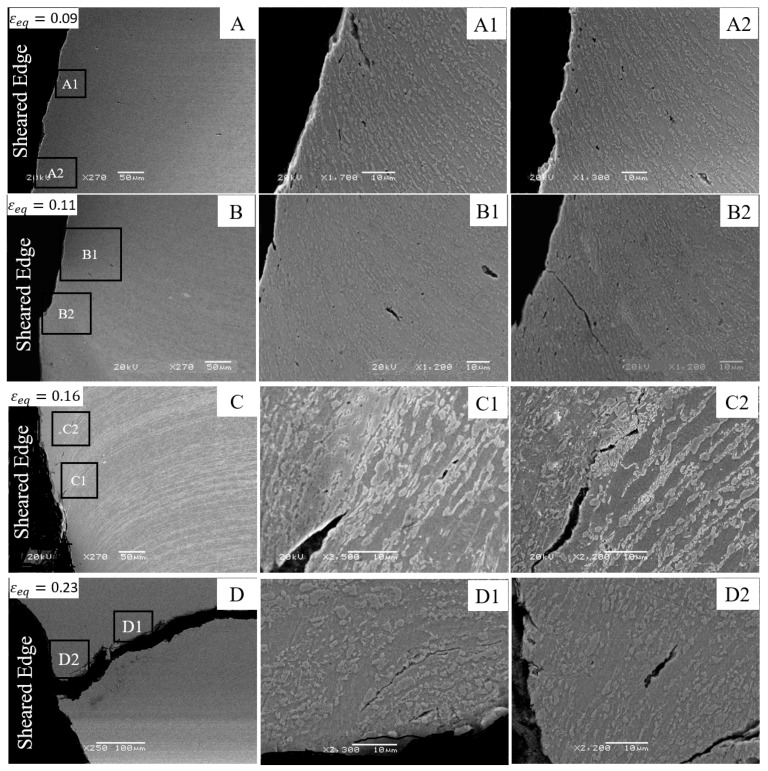
SEM micrographs of the interrupted DP780 sheared edge hole tension specimens at different strain levels: (**A**) 0.09; (**B**) 0.11; (**C**) 0.16; and (**D**) 0.23.

SEM micrographs of the DP780 sheared edge at the different strain levels are shown in Figure 12. During the shearing operation, grains have a tendency to orient along the shearing direction and the rotated grain boundaries are referred to herein as shear flow lines. Micrograph A, obtained at the strain level of 0.09, represents the early stage of damage initiation. Similar to the DP780 reamed edge, void nucleation due to the fracture of martensite islands is the primary void nucleation mechanism away from the DP780 sheared edge (as shown in micrographs A1 and A2). The crack initiates at the sheared edge at a lower strain of 0.09, as indicated in magnified image A1. The image A2 indicates a series of voids nucleated at martensite islands. Additional cracks form behind the sheared edge through coalescence of voids, as seen in micrograph B2. A higher magnification of the crack tip in micrograph C1 reveals void formation along the crack front. Void growth and crack propagation tend to occur along the shear flow lines, as shown in the magnified image C2, which ultimately causes failure as shown in micrograph D. The banded distribution of martensite results in cracking and debonding along the interface (seen in micrograph D1). Investigation of the morphology of the crack edge reveals a zig-zag crack pattern on the failed DP780 sheared edge. This zig-zag pattern of the crack surface is a characteristic of ductile failure and suggests a ductile fracture mode in the DP780 sheared edge.

### 4.2. Fracture Mechanism in CP800

**Figure 13:**
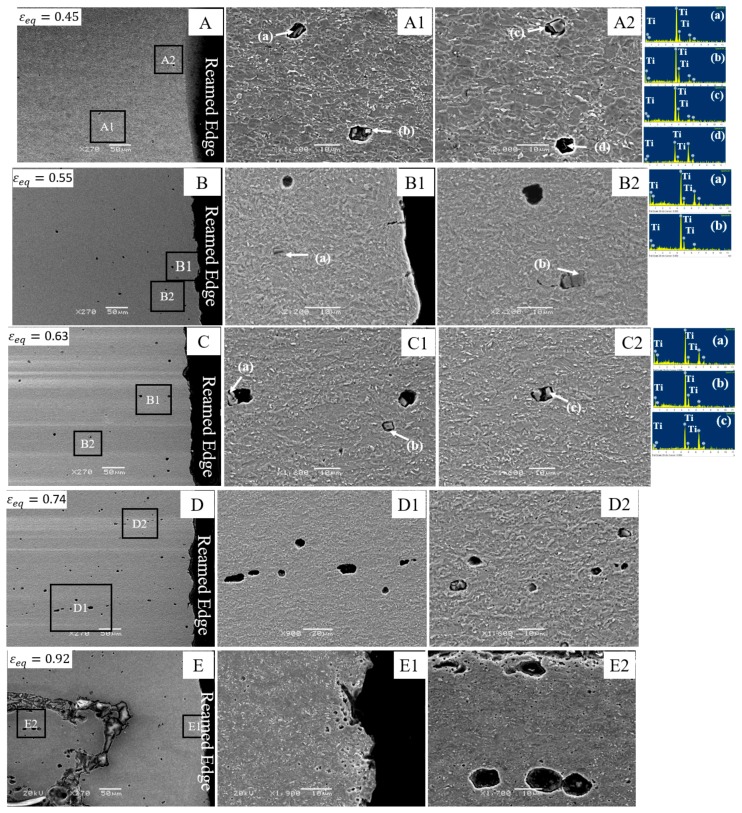
SEM micrographs of the interrupted CP800 reamed edge hole tension specimens at different strain levels: (**A**) 0.45; (**B**) 0.55; (**C**) 0.63; (**D**) 0.74; and (**E**) 0.92.

Figure 13 serves to show the damage development in the CP800 during reamed edge stretching. Micrographs A, B, C, D, and E were acquired from the samples interrupted at different strain levels: 0.45, 0.55, 0.63, 0.74, and 0.92 (fracture), respectively. The nucleated voids seen in micrograph A are shown at a higher magnification in images (A1 and A2). The EDS spectra acquired on the four cavities (a, b, c, and d) reveals that titanium nitride inclusions were the main nucleation sites in the CP800 steel. An enlarged image of the EDS spectrum for cavity (a) is shown for clarification. The void nucleation at particles does not occur simultaneously for particles of different size and the nucleation strain appears dependent upon the particle size and shape [29]. For instance, the small-sized particles shown in micrograph B1 do not nucleate, however, void formation initiates at a larger particle as shown in micrograph B2 indicating that nucleation strain is higher for smaller particles [42]. Erdogan et al. [29] reported that an inclusion with higher aspect ratio can undergo multiple internal fractures as seen in enlarged image B2. The micrographs (C1 and C2) acquired at 0.63 strain demonstrate two different void formation mechanisms associated with inclusions in the CP800 steel. Cavity formation at inclusions occurs either due to the de-bonding of the TiN particles from the matrix, as shown in magnified image C1, or fracture of the inclusion as observed in image C2. The magnified images D1 and D2 acquired at a strain level 0.73 demonstrate the formation of primary voids associated with inclusions in addition to secondary voids nucleated at bainite or martensite interfaces. The majority of the primary voids are most likely associated with titanium nitride large particles. At higher strain, martensite and bainite particles either separate from the matrix or fracture due to the severe deformation and nucleate secondary voids. The growth of primary voids is evident in micrograph D1. The edge of the fractured CP800 reamed sample is shown in micrograph E. Near failure, the rate of formation of secondary voids rapidly increases as shown in micrograph E1. A typical void impingement coalescence is shown in magnified image E2 and indicates a ductile failure mechanism for the CP800 reamed edge.

**Figure 14:**
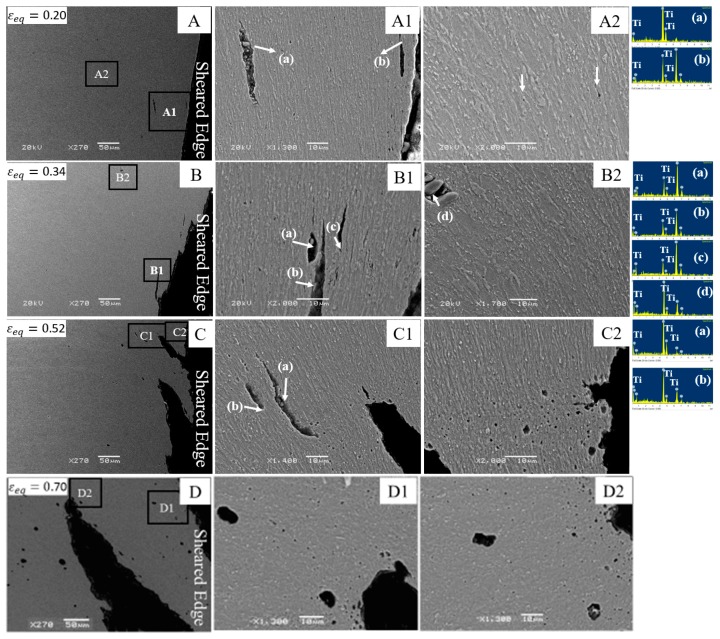
SEM micrographs of the interrupted CP800 reamed edge hole tension specimens at different strain levels: (**A**) 0.20; (**B**) 0.34; (**C**) 0.52; and (**D**) 0.70.

In order to investigate the fracture mechanism near the CP800 sheared edge, SEM examination was conducted on the hole tension specimens interrupted at four different strain levels, shown in Figure 14. Micrographs A, B, C, and D show the advancement of damage beneath the sheared edge at strain levels of 0.20, 0.34, 0.52, and the failure strain 0.70, respectively. Early damage in the form of void growth behind the sheared edge is shown in micrograph A, followed by formation of a crack at the edge indicated in micrograph B. Further deformation results in formation of multiple cracks along with growth of the existing cracks (shown in micrograph C) and consequently results in failure as observed in micrograph D. Similar to the reamed edge, primary voids are associated with inclusions behind the sheared edge. The enlarged image A1 of micrograph A acquired near the edge shows the voids nucleated by cracking of large TiN particles. The presence of TiN particles is evident from the EDS spectrum. The secondary voids nucleated at bainite/martensite interfaces located away from the edge are shown in image A2. During further deformation, primary voids begin to nucleate away from the edge at TiN particles as shown in image B2. Near the crack tip, a cluster of voids is observed as shown in micrograph B1. Subsequent growth of the crack tip occurs by interaction of multiple voids with the crack along the crack front. This mechanism leads to propagation of the crack along the direction of flow lines, as shown in micrograph C. The enhanced nucleation and growth of voids near the crack-tip is evident in micrographs C1 and C2 and ultimately causes failure, as seen in micrograph D. The characteristic appearance of a ductile fracture is demonstrated beneath the CP800 sheared edge in micrographs D1 and D2.

## 5. Quantitative Examination of Damage Progression

**Figure 15:**
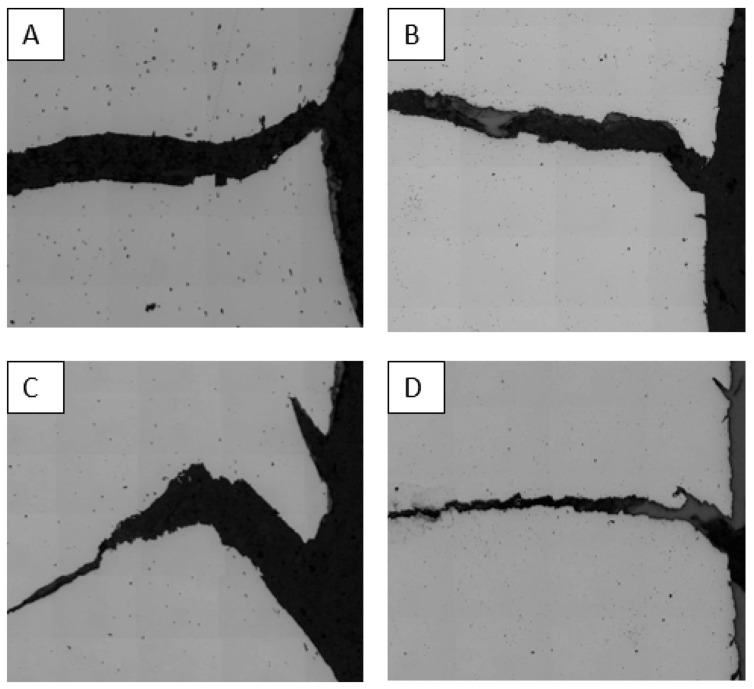
In-plane optical micrographs of the fractured (**A**) CP800 reamed; (**B**) DP780 reamed; (**C**) CP800 sheared; and (**D**) DP7800 sheared edge hole tension specimens.

The SEM observations conducted on the sheared and reamed holes at the different strain levels indicate ductile failure modes for both the DP780 and CP800 steels. Figure 15 shows the in-plane micrographs of the fractured specimens of the DP780 and CP800 steels with both the edge conditions and indicates typical ductile crack propagation. In the following sections, quantitative analysis for each stage of ductile failure, void nucleation, growth and coalescence, is conducted for the two steels. The effect of the two initial microstructures, as well as their sensitivity to shearing, on damage accumulation is discussed by comparing the void evolution rates for each condition.

### 5.1. Void Nucleation

**Figure 16:**
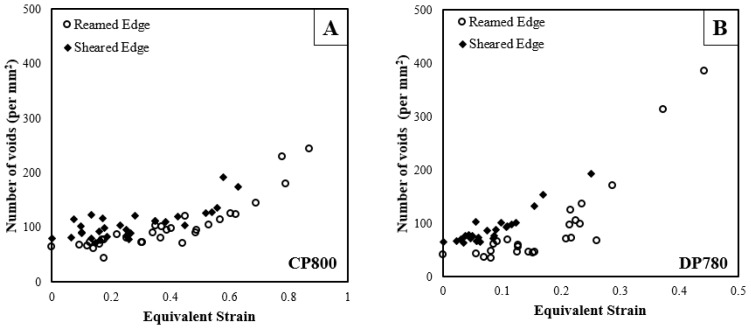
Evolution of void density during hole tension tests on (**A**) CP800; and (**B**) DP780.

Quantitative porosity measurements were performed on the interrupted hole tension samples to compare damage progression in the CP and DP steels during stretching of the reamed and sheared edges. The number of voids per unit area as a function of equivalent strain obtained from the DIC measurements is plotted in Figure 16. Each point on the curve corresponds to a different AOI of the interrupted samples, as indicated in Figure 10. The extent of void nucleation increases with the equivalent strain for both edge conditions and materials.

In addition to the void density, various void measurements such as void area fraction, average diameter, angle of orientation, and void aspect ratio were acquired on each AOI of the sample, as presented in the following sections. In order to analyze the damage evolution effectively, measured data was aggregated by binning into fixed strain intervals of 0.05 and 0.10 for the DP780 and CP800, respectively.

**Figure 17:**
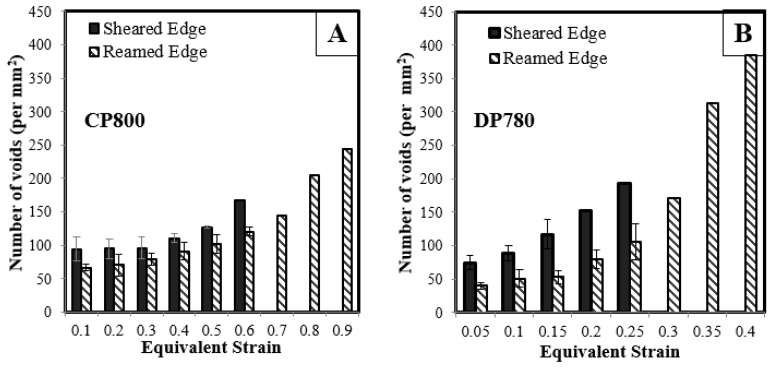
Average void density versus equivalent strain for (**A**) CP800; and (**B**) DP780. Note that the number of measurements at higher strain drops to unity so that a standard deviation cannot be calculated (no scatter bands).

Figure 17 shows the increase in numbers of voids (per unit section area) with increased equivalent strain using this binning technique for both materials and edge conditions. The number of data points acquired at a particular strain varies, with a higher number of data points aggregated at a lower strain and decreases to one data point near the failure strain. The standard deviation associated with aggregate quantity of nucleated voids for the two edge conditions and the two materials is in the range of 4–16 void/mm^2^, except at large strain, for which there is a single data point. The increase in numbers of voids with strain indicates that nucleation occurs continuously until failure, with higher numbers of voids at higher strain, as seen in Figure 13.

The CP800 microstructure exhibits lower numbers of nucleated voids compared to DP780 for a given strain. The void density for the CP800 reamed edge is 91 per mm^2^ at 0.4 strain while the void density for DP780 is four times higher at 385 per mm^2^. At lower strains, this difference is less pronounced which suggests a high nucleation rate within the DP780 microstructure. The number of nucleated voids at a particular strain level is observed to be higher for the sheared edge compared to the reamed edge for both the steels. At 0.60 strain, the number of voids is 167 and 122 per mm^2^ for the CP800 sheared and reamed edges respectively. Similarly for the DP780, the quantity of nucleated cavities at 0.25 strain is 106 and 192 per mm^2^ for the reamed and sheared edges respectively.

### 5.2. Void Growth and Evolution

**Figure 18:**
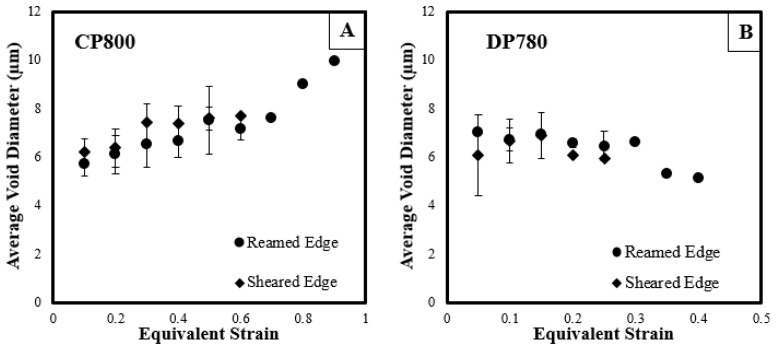
The variation of average void size during the deformation of (**A**) CP800; and (**B**) DP780.

Figure 18 shows the average equivalent diameter of voids behind the reamed and sheared edges. The average void diameter increases with strain for the CP800 steel. The DP780 however has an almost constant average void diameter for the entire deformation history, except just prior to failure at which point the average diameter decreases. A similar observation was reported by Avramovic-Cingara et al. [43] and Landron et al. [44] for a DP600 steel. This phenomenon could be attributed to the continuous nucleation of smaller voids during the deformation process. The primary (large) voids are growing and coalescing into larger voids but the nucleation of small voids is leading to a near constant average void size. Collectively, the average void size remains almost constant until just before failure at which point a higher rate of nucleation of smaller voids associated with final failure reduces the overall average diameter. The variation of average void diameter during the deformation is similar for the reamed and sheared edges and suggests that the edge condition has little influence on the void size.

**Figure 19:**
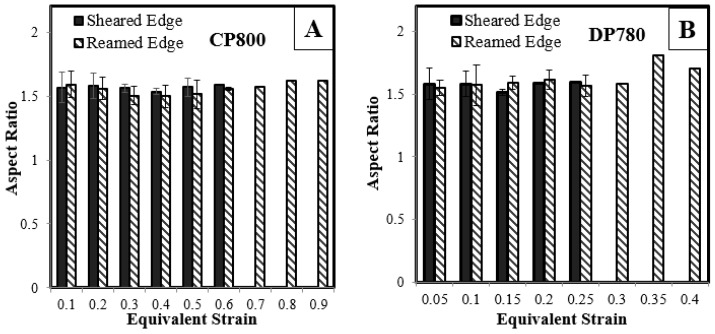
Variation of the average void aspect ratio for (**A**) CP800; and (**B**) DP780.

The void aspect ratio is defined as the ratio of the major to minor void diameters. The initial value of aspect ratio is dependent on the aspect ratio of nucleating particles that are initially aligned in the rolling direction. During deformation, voids grow in the loading direction that alters the aspect ratio with the variation in void shape due to deformation shown in Figure 19 for both the materials and edge conditions which remain nearly constant. The aspect ratio of individual voids is expected to evolve with the deformation but the average void aspect ratio remains unaffected during the deformation due to the continuous nucleation of voids.

**Figure 20:**
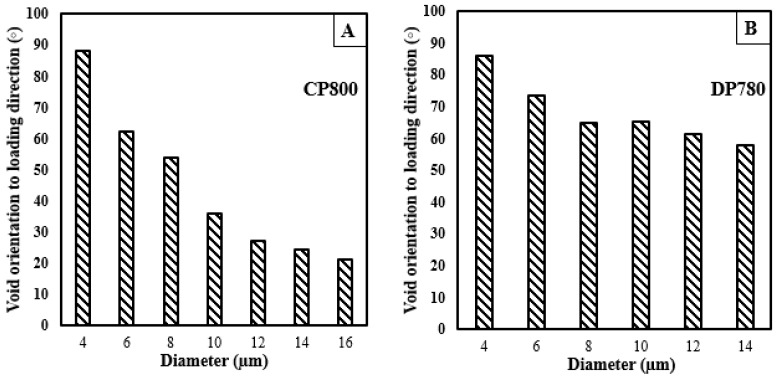
Void orientation relative to the loading direction as a function of void diameter for the (**A**) CP800 and (**B**) DP780 reamed edges.

**Figure 21:**
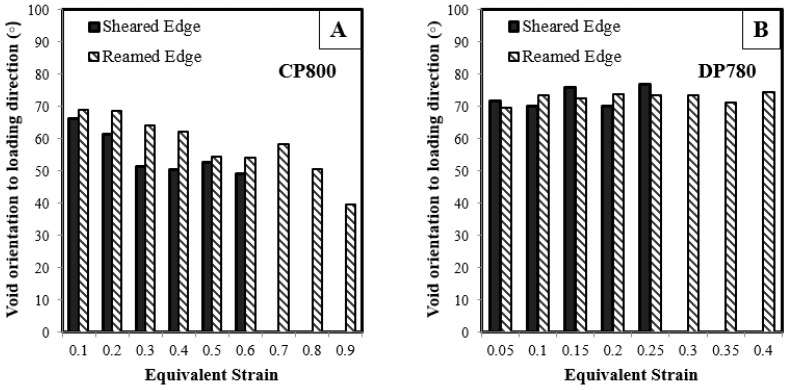
Void orientation relative to the loading direction as a function of equivalent strain for the (**A**) CP800; and (**B**) DP780.

Initially, voids can be aligned randomly in any direction. During deformation, voids tend to grow along the principal loading axes such that the void orientation becomes a function of void size as shown in Figure 20. The orientation of a void is defined as the angle of the major void axis with respect to the principal loading direction. Thomason et al. [45] stated that the orientation of a particle with respect to the direction of principal loading significantly influences damage evolution. The void orientation with respect to loading direction as a function of strain is shown in Figure 21. Since the average void diameter of the entire void population remains almost constant for DP780, the average orientation angle remains constant. In the CP800, a slight increase in void diameter is observed at higher strain, therefore the average void orientation decreases from 70° to 47°. The void rotation behind the sheared and reamed edges follows similar trends for both the CP800 and DP780 steels. The SEM analysis conducted along the through-thickness direction in the previous section indicated that unlike the reamed edge, the voids grow along the shear flow lines behind the sheared edge. The difference in the void orientation was not captured because the void measurement was conducted on the in-plane orientation of the hole tension specimen. Future work will investigate the influence of shearing on the void orientation using 3D tomography.

### 5.3. Damage Accumulation

**Figure 22:**
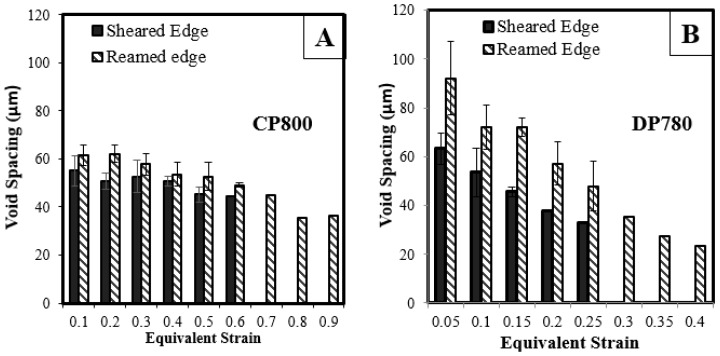
Variation of the void spacing with equivalent strain for (**A**) CP800; and (**B**) DP780.

Void coalescence is the final stage of ductile failure and occurs through the localization of the ligament between neighboring voids [46]. Thomason [47] proposed a criterion for the onset of localization between voids and showed that coalescence depends strongly on the void aspect ratio and the relative void spacing, defined as the ratio of the length of the ligament connecting nearest neighboring voids to the lateral radius. The plot of void spacing with respect to strain for the CP800 and DP780 sheet is presented in Figure 22 for the reamed and sheared edges. The void spacing was calculated as a center-to-center distance between the nearest neighboring voids. With an increase in strain, the void population increases leading to formation of voids at a shorter distance and therefore the void spacing decreases as deformation progresses. The initial void spacing behind the sheared edge is lower compared to the reamed edge for both steels.

**Figure 23:**
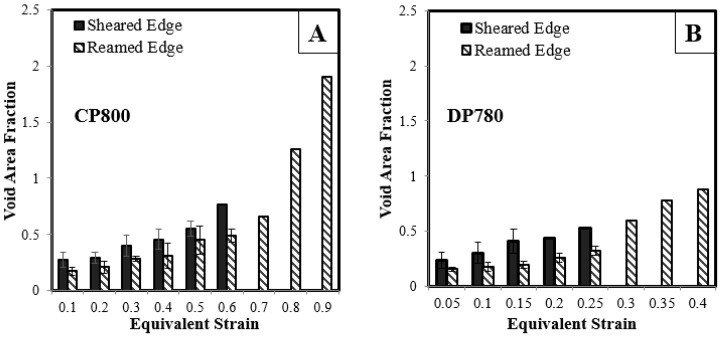
Evolution of the void area fraction with respect to equivalent strain for (**A**) CP800; and (**B**) DP780.

The void area fraction (porosity) is equivalent to the total area of voids divided by the total section area considered. Figure 23 shows the void area fraction versus equivalent strain for the two materials. As a material deforms, the number of void cavities increases, the existing voids grow and coalescence initiates prior to the onset of fracture. This damage evolution results in a continuous increase in the void area fraction as deformation progresses. The porosity is higher for the DP780 compared to the CP800. At failure, the void area fraction for the sheared edges is lower than for the corresponding reamed edges revealing that the sheared edge fails at a lower porosity. This observation verifies that the damage accumulation is higher for the ferritic-martensitic DP780 microstructure than the ferritic-bainitic CP800 for a particular strain.

## 6. Modeling

**Figure 24:**
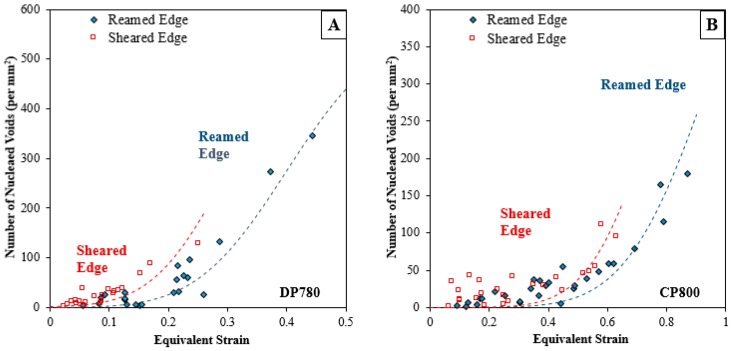
The implementation of Chu and Needleman’s nucleation rule using Equation (5) for the (**A**) CP800 and (**B**) DP780 steels.

The experimental observations in the previous sections demonstrate that damage development behind the reamed and sheared edges is progressive in the CP800 and DP780 steels. An appropriate model for each stage of the ductile fracture process (i.e., void nucleation, growth, and coalescence) should be able to capture damage evolution analytically. Chu and Needleman [32] developed a strain-controlled nucleation criterion, assuming the strain required to nucleate a void follows a normal distribution and is expressed as

(5) $$\dot{N}=\frac{N_{n}}{s_{N}\sqrt{2\pi }}exp\left[-\frac{1}{2}{\left(\frac{\varepsilon^{p}-\varepsilon_{N}}{s_{N}}\right)}^{2}\right]{\dot{\varepsilon }}_{p}$$

where $\dot{N}$ is the void nucleation rate, *N_n_* is the maximum number of voids per unit area available to nucleation voids, *ε_N_* and *s_N_* are the mean and standard deviation of the nucleation strain, and *ε_p_* is the Von Mises equivalent plastic strain. The SEM investigation of the CP800 and DP780 microstructures indicates that void nucleation occurs at the ferrite-martensite or ferrite-bainite interfaces and at the martensite or TiN particles. Since nucleation occurs at random locations along the interfaces or within the individual phases, the maximum number of void nucleation sites in the CP and DP steels is difficult to measure, making the determination of *N_n_* from metallurgical analysis challenging. Hence, values of *N_n_* were taken as 700 mm^−2^ and 350 mm^−2^ for the DP780 and CP800 materials, respectively, after which the parameters in Equation (5) were fit using the MATLAB dfittool for normal distribution to the observed void point fraction versus strain histories in Figure 16. The resulting fits are shown in Figure 24 and capture the observed void point fractions relatively well. Table 4 summarizes the nucleation parameter fits for each material and edge condition. The average nucleation strains are lower for the sheared edge condition, reflecting the prior deforming during the shearing process. Note that the value of *N_n_* was assumed to be the same for the reamed and sheared edge conditions since the maximum number of nucleation sites is a microstructure-dependent property.

**Figure 25:**
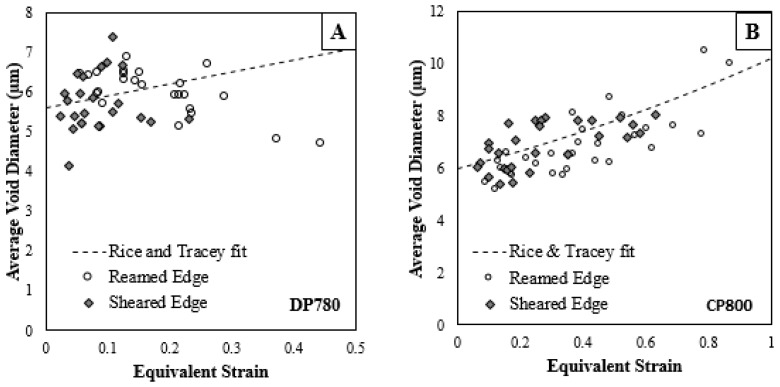
Evolution of void growth using Equation (6) for reamed and sheared edges of the (**A**) CP800 and (**B**) DP780 steels.

Huang [48] modified Rice and Tracey [33] model to evaluate the growth of spherical voids in an infinite, rigid, ideally plastic Von Mises material, expressed as:

(6) $$\frac{dR}{R}=\alpha \;exp\left(\frac{3}{2}T\right)d\varepsilon$$

where *R* is the initial radius of the cavity, *T* is stress-triaxiality, ε is the equivalent plastic strain, and α is the material constant. The void aspect ratio remains essentially constant with a value of approximately 1.6 for both the reamed and sheared edge conditions (as presented in Figure 19A,B for the CP800 and DP780 respectively). To simplify modeling, the voids were assumed to be spherical in the current model and the average void diameter for the CP800 and DP780 was predicted using Equation (6) and is presented in Figure 25. The initial void diameter was assumed to be 6 μm and 5.6 μm for CP800 and DP780, respectively. In general, the experimental trend of increasing void size with strain is captured well for the CP800 data. For DP780, the measured average void size actually decreases with strain due to nucleation of smaller voids at later stages of deformation. This phenomenon could not be captured by the simple void growth model used in the current study. A model (and experimental measurements) distinguishing between multiple void populations would be required to capture such behavior and was beyond the current measurement capability which uses interrupted specimens as opposed to in situ measurements tracking each void.

The plastic limit-load (PLL) coalescence model of Thomason [47] has replaced the traditional critical porosity model of Tvergaard and Needleman [49] as coalescence is a natural consequence of the stress state and geometry of the microstructure. The void coalescence mechanism in the PLL model is due to necking failure of the inter-void ligament, which occurs transverse to the principal loading direction. Several extensions of Thomason’s PLL model have been proposed [34,50,51], and have been shown to give excellent agreement with unit cell computations. Recently, Benzerga and Leblond [34] obtained the first closed-form analytical solution for void coalescence by internal necking in a perfectly plastic material where coalescence occurs when

(7) $$\frac{\sigma_{b}}{\sigma_{y}}\geq C_{f}\left(\chi ,\;W\right)=\frac{1}{\sqrt{3}}\left[2-\sqrt{1+3\chi^{4}}+\ln\frac{1+3\chi^{4}}{3\chi^{2}}\right]+\frac{1}{3\sqrt{3}}\left[\frac{\chi^{3}-3\chi +2}{\chi W}\right]$$

where *σ_b_* is the maximum principal stress, *σ_y_* is the yield stress, *W* is the void aspect ratio, and *χ* is the ligament size ratio defined as the ratio of the lateral void radius, *R_x_*, to the lateral void spacing, *L_x_*, for a periodic arrangement of 3-D unit cells [52]. The above equation states that void coalescence begins when the ratio of *σ_b_* and *σ_y_* reaches a critical value, *C_f_*. The stress-bearing capacity *σ_b_* decreases when the voids are closely spaced i.e., *χ* decreases.

The average value of void spacing, *L* was calculated from the measured number of voids in unit area, *N*, with an assumption that voids were homogeneously distributed [53]:

(8) $$L=1/2N^{-0.5}$$

**Figure 26:**
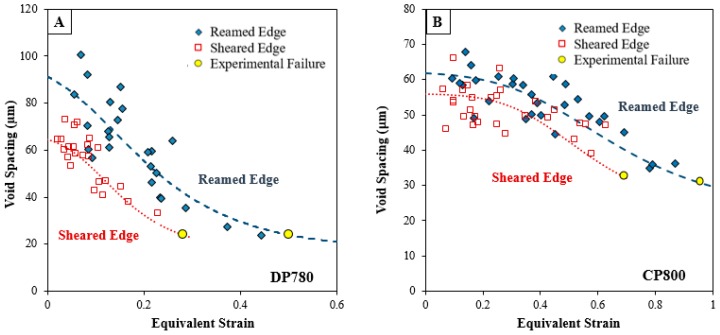
The evolution of void spacing ratio for the reamed and sheared edges of the (**A**) CP800 and (**B**) DP780 calculated analytically using Equation (8).

**Figure 27:**
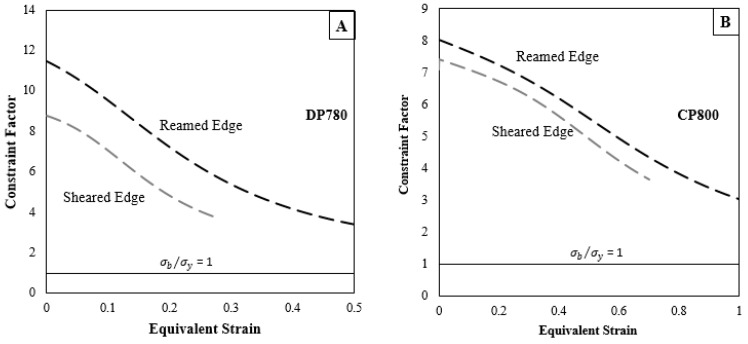
Constraint factor for the reamed and sheared edges of the (**A**) CP800 and (**B**) DP780 steels using Equation (7).

The void spacing ratio as a function of strain was obtained for each condition using Equations (6) and (8) to predict *R_x_* and *L_x_* respectively and shown in Figure 26. The evolution of constraint factor as a function of strain for the two edge conditions is compared for the two materials using Benzerga and Leblond’s criterion in Figure 27. The constraint factor for the reamed edge is higher than for the sheared edge for both materials due to a higher nucleation rate within the SAZ that influences void spacing ratio. Consequently, the onset of localization is predicted at a lower strain behind the sheared edge than the reamed edge.

In cases where no necking is observed in a material i.e., assuming homogeneous deformation throughout the deformation, the ratio of *σ_b_* and *σ_y_* is unity at coalescence. The constraint factors indicated in Figure 27 for all edge conditions exceed unity and consequently overestimate failure strain because, in reality, the material necks during deformation at which point the ratio of *σ_b_* and *σ_y_* would increase. In addition, local particle cluster will affect spacing, whereas the current model uses average values. The analytical estimation of stress-state around a void is challenging and therefore requires finite element modeling. Future work will focus on developing a damage-based material model to predict failure strain and capture necking using finite element simulation of a hole tension test.

## 7. Discussion

### 7.1. Effect of Microstructure Constituents on Damage Mechanism

The SEM investigation on DP780 revealed four kinds of nucleation mechanism: (1) fracture of martensite; (2) decohesion of ferrite-martensite interfaces; (3) ferrite grain failure; and (4) cavity formation at TiN particles. The former two were the most prominent nucleation mechanisms observed in DP780, in contrast with the CP800 steel for which the TiN particles are the main source of void nucleation. Zhang et al. [30] observed that when the martensite fraction was high, localized failure strain was low due to rapid nucleation of voids. Additionally, Erdogan et al. [29] reported that blocky martensite is more susceptible to cracking than fine martensite islands due to the stress concentration and dislocation pile-ups on blocky particles that induce cracking. Since the DP780 is comprised of a higher fraction of interconnected martensite, cracking of the martensite occurs more easily in DP780 compared to CP800. The primary source of void nucleation in the CP800, however, is nucleation at TiN particles and secondary void formation at martensite/bainite particles near failure strain. Bainite, as a microstructural constituent, is not as strong as martensite, so to match the tensile strength of DP780, different techniques of strengthening are required in the CP800. The presence of a small fraction of martensite provides some contribution in imparting strength to the CP800 steel. The strength of CP800 is mainly governed by the addition of Ti for precipitation strengthening and solid solution strengthening [6]. Consequently, the fraction of TiN particles is higher in the CP800 steel compared to the DP780 steel and is responsible for primary void nucleation in the CP800 steel.

**Figure 28:**
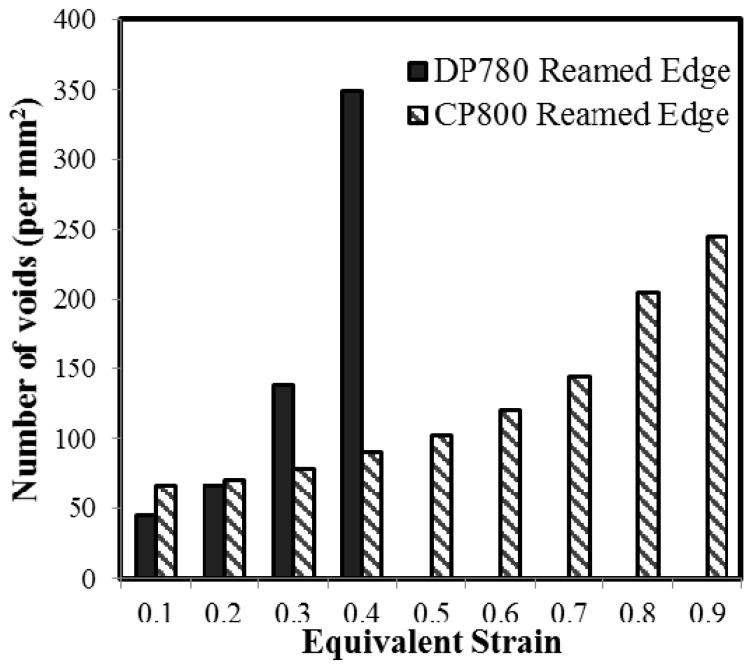
The number of voids per unit area for the reamed and sheared edges of the CP800 and DP780 steels.

The resistance to deformation of martensite is significantly higher than that of ferrite due to the strength mismatch. Typically, bainite manifests as ferrite plates separated by martensite, retained austenite or cementite [5]. Bainite therefore has a tendency to deform more easily than martensite. Previous work reported improved edge formability in the ferritic-bainitic DP steel compared to the ferritic-martensitic DP steel. Ballinger and Gladman [54] suggested that strain partitioning between bainite and ferrite is lower compared to that between martensite and ferrite. TEM examination conducted by Saha et al. [55] suggested that a high dislocation density exists in the ferrite adjacent to martensite compared to the ferrite adjoining bainitic-areas. The distribution of nucleation sites is ultimately dependent on the composition, distribution, strength, and interaction of different phases in a material. Since the fraction of martensite in DP780 is considerably more than the quantity of inclusions in the CP800, the number of favorable sites for void nucleation is significantly higher in the DP780 compared to the CP800. Secondly, the strength differential of martensite-ferrite interfaces is higher than that of the ferrite-bainite interface; therefore, voids nucleate at a lower strain in the DP780. Additionally, the DP780 has around 40% martensite and therefore a huge number of interfaces with a strong phase differential is present to nucleate voids. The CP800 has mainly a lower fraction of TiN particles so fewer sites for nucleation are available until very large strain which triggers bainite void nucleation. These properties result in a remarkably higher rate of void nucleation in the DP780 that causes failure at a lower strain and consequently results in poor edge stretchability for the DP780 compared to the CP800 as shown in Figure 28.

### 7.2. Effect of Edge Condition on Damage Mechanism

The nucleation mechanism for a particular alloy did not change with edge condition; the effect of shearing was to locally harden the edge which in turn increased the rate of nucleation. The fracture strain for the sheared edge is lower than the reamed edge for both materials investigated in this study. For the DP780 sheared edge, the failure strain is 0.24 which is essentially one-half of the failure strain reported for the reamed edge (0.50). The failure strains for the CP800 sheared and reamed edges are 0.60 and 0.90, respectively. Interestingly, the CP800 steel is less sensitive to shearing and therefore the percentage reduction in failure strain between the sheared and reamed edges is 50% for the DP780 versus 33% for the CP800. The amount of pre-straining and damage introduced during the shearing process depends on the microstructural constituents. Since the ferritic-bainitic material offers less strength differential than the DP steels, the work-hardening is lower at the CP800 sheared edge compared to the DP780 [15,16,18]. This difference in the level of work-hardening alters damage evolution and, therefore, CP800 provides better performance in sheared edge stretching than DP780 steels.

Another factor that could result in differences in damage evolution within the various alloys and hole edge conditions is the residual stress generated during drilling versus shearing operations. The measurement of residual stress profiles and their influence on damage development was not considered in the current study but should be considered in future work.

The analytical model presented in this work indicates that to predict sheared edge failure it is critical to account for the difference in the rate of nucleation behind the two edge conditions for both materials. The evolution of constraint factor as a function of the two edge conditions (shown in Figure 28) indicates that the void spacing affects the resistance to localization and a smaller void spacing behind the sheared edge resulted in onset of void coalescence at a lower strain. Future work will focus on developing a damage-based model that can account for the difference in damage evolution behind the two edge conditions.

## 8. Conclusions

(1) Comparison of the limit strains in the current hole tension experiments and the hole expansion results presented by Pathak et al. [7] indicates that the edge limit strains are the same, at least for the current materials and hole processing methods.
(2) In the DP780 steel examined herein, martensite cracking and decohesion of ferrite-martensite interfaces are the dominant nucleation mechanisms. A smaller quantity of voids is nucleated due to failure of ferrite grains near the fracture strain. Voids associated with TiN particles were few and formed at a lower strain.
(3) Two nucleation mechanisms related to TiN particles were observed in the CP800 steel: inclusion cracking and decohesion. The primary voids were related to TiN particles, while secondary voids nucleated within the martensite or bainite microstructures at larger strains.
(4) The microstructure of DP780 consists of a higher fraction of martensite that has a larger strength differential with the ferrite matrix, resulting in a lower nucleation strain and accelerated void nucleation in comparison to the ferritic-bainitic CP800 steel that has a lower strength differential between phases.
(5) The nucleation mechanism observed behind the sheared edge is similar to the reamed edge for both materials investigated in this study.
(6) The rate of damage accumulation is higher behind the sheared edge relative to the reamed edge due to the presence of pre-straining behind the sheared edge that promotes nucleation, growth, and coalescence of voids.
(7) Once a crack initiates at the sheared edge, crack propagation occurs continuously ahead of the crack-tip though accelerated void nucleation, growth, and coalescence and ultimately causes failure.
(8) The Chu and Needleman criterion for nucleation, the Rice and Tracey criterion for void growth and the Benzerga and Leblond criterion for coalescence were adopted in the present work to account for the variation in damage progression between the reamed and sheared edges. The quantitative data from experiments were closely predicted by the analytical models and suggests that a damage-based fracture model can be developed based on the void evolution during the hole tension test to predict edge failure.

## Figures and Tables

**Table 1:** The percentage of chemical compositions of the CP800 and DP780 steels.

Material	C	Mn	Si	Cr	Ti
CP800	0.05	1.5	0.55	0.6	0.14
DP780	0.14	2.0	0.26	0.26	0.023

**Table 2:** Mechanical properties of the CP and DP steels. The value in the brackets is the standard deviation after three tests [7].

Material	Thickness (mm)	Direction	Yield Strength (MPa)	Ultimate Tensile Strength (MPa)	Total Elongation (%)	*n* (5-UE%)	*r*	*r_n_*	*r_a_*	Reduction of Area (%)
CP800	2.90	RD	710	810	19.6	0.08	0.70	1.08	−0.51	69 (6)
			(6)	(3)	(1.7)	(0.00)	(0.04)			
		TD	788	850	18.8	0.06	0.95			
			(5)	(5)	(1.0)	(0.00)	(0.04)			
		DD	726	800	20.5	0.07	1.33			
			(8)	(5)	(2.0)	(0.00)	(0.03)			
DP780	1.56	RD	509	800	22.8	0.16	0.72	0.90	−0.16	41 (4)
			(8)	(6)	(2.2)	(0.00)	(0.02)			
		TD	522	806	21.6	0.15	0.92			
			(4)	(5)	(1.8)	(0.00)	(0.03)			
		DD	533	815	25.5	0.15	0.98			
			(6)	(8)	(1.8)	(0.00)	(0.01)			

**Table 3:** Equivalent failure strain and displacement to failure for the reamed and sheared holes of the CP800 and DP780 steels during the hole tension test.

Materials	Edge Condition	Equivalent Failure Strain	Displacement to Failure (mm)				
			1	2	3	4	Failure
CP800	Reamed	1.01 ± 0.04	1.78	1.89	1.97	2.31	2.42
	Sheared	0.66 ± 0.04	0.91	1.37	1.59	1.75	1.81
DP780	Reamed	0.54 ± 0.03	1.06	1.15	1.31	-	1.51
	Sheared	0.28 ± 0.04	0.53	0.60	0.66	-	0.82

**Table 4:** Nucleation parameters for the reamed and sheared holes of the CP800 and DP780 steels indicated in Equation (5).

Material	Edge Condition	*N_n_* (per mm^2^)	$\varepsilon_{N}$	$s_{N}$
CP800	Reamed	350	0.85	0.23
	Sheared	350	0.70	0.19
DP780	Reamed	700	0.42	0.12
	Sheared	700	0.30	0.10
